# Structure and Wear Resistance of TiC-Reinforced Al_1.8_CrCuFeNi_2_ High-Entropy Alloy Coating Using Laser Cladding

**DOI:** 10.3390/ma16093422

**Published:** 2023-04-27

**Authors:** Jing Guo, Chenghao Liu, Dexing Wang, Lingfeng Xu, Kaikai Song, Ming Gao

**Affiliations:** 1 College of Mechanical and Electronic Engineering, Shandong Agricultural University, Tai’an 271018, China; gjcp@sdau.edu.cn (J.G.); lch1996721@163.com (C.L.); 17861501557@163.com (D.W.); 2 Shandong Provincial Key Laboratory of Horticultural Machineries and Equipments, Tai’an 271018, China; 3 Shandong Provincial Engineering Laboratory of Agriculture Equipments Intelligence, Tai’an 271018, China; 4 School of Mechanical, Electrical and Information Engineering, Shandong University, Weihai 264209, China; songkaikai8297@gmail.com

**Keywords:** high-entropy alloys, wear resistance, coatings, titanium carbide

## Abstract

Al_1.8_CrCuFeNi_2_ high-entropy alloy coatings with different TiC contents were prepared using laser cladding. The effect of TiC on the microstructure, hardness and wear resistance of the coatings was investigated. It was found that the phase structure of the coating with 10 wt.% TiC was a single BCC phase with no other precipitated phase. When 20 wt.% TiC was added, the phase structure of the coating was a BCC phase and TiC phase. When the TiC content increased to 30 wt.%, more TiC-reinforcing phase was formed. With the increase in the TiC content, the hardness of the high-entropy alloy coating was enhanced and the wear loss clearly decreased, which was closely related to the change in the coating structure. The addition of TiC to high-entropy alloys plays the role of fine-grain strengthening and dispersion strengthening.

## 1. Introduction

Laser cladding is an additive manufacturing technology that can be used to optimize the quality and performance of the workpiece surface. With the continuous development of laser and mechanical technology, the application of laser cladding is more and more extensive [1,2]. In order to improve the wear resistance of common parts, a layer of material with better wear resistance is often prepared on the parts using laser cladding technology.

High-entropy alloys (HEAs) with high hardness, outstanding wear resistance and excellent corrosion resistance [3,4,5] are considered as promising materials in the fields of high-temperature-resistant alloys, wear-resistant alloys and corrosion-resistant alloys, etc. [6,7]. Therefore, HEAs can be used as wear-resistant coating layers. Some investigations on laser cladding high-entropy alloy coatings have been reported. The hardness and wear resistance of the coatings can be improved using laser cladding TiZrAlNb and TiZrAlNbCo high-entropy alloys on a TC4 substrate. The Al_1.8_CrCuFeNi_2_ HEA is a typical high-entropy alloy with excellent wear resistance and a high hardness [8]. It is very suitable as a coating layer to improve the wear resistance of the substrate.

Relevant reports show that a change in elements or the addition of strengthening particles will improve the strength and wear resistance of materials. Changes in the carbon content also affect the quality of the laser cladding coating [9,10,11]. In order to improve the wear resistance of the coating, some reinforced particles are added to the high-entropy alloys, so as to form a new phase with greatly improved hardness and wear resistance. Generally, the hardness and wear resistance of ceramic materials are better than those of high-entropy alloys [12]. Ceramic materials are also a hot spot in the research on strength plasticity matching in high-entropy alloy systems in recent years [13]. Some ceramic particles are chosen to form excellent composite coating alloy systems. At present, the types of ceramic-reinforced particles added to alloy systems mainly include carbide ceramics, nitride ceramics, oxide ceramics, nickelide ceramics, composite ceramics and natural ceramics [14,15], such as WC, TiC, SiC in carbides and Si3N4, BN, AlN in nitrides, etc. In particular, TiC ceramic particles have shown excellent strengthening effects in related reports [16,17,18,19].

The purpose of this study was to obtain materials with better wear resistance. In this study, TiC particles with 10 wt.%, 20 wt.% and 30 wt.% content were added into the Al_1.8_CrCuFeNi_2_ high-entropy alloy powder, and then the laser cladding experiment was carried out. The effect of TiC on the microstructure, hardness and the wear resistance of the Al_1.8_CrCuFeNi_2_ high-entropy alloy coating prepared using laser cladding was analyzed.

## 2. Materials and Methods

### 2.1. Preparation of TiC-Reinforced High-Entropy Alloy Coating

Due to the excellent strength and hardness of 65 Mn high-carbon spring steel, it has become the main material for wear-resistant parts. In this study, 65 Mn spring steel was selected as the matrix material to investigate the wear resistance of the coating.

The Al_1.8_CrCuFeNi_2_ HEA exhibited high hardness and excellent wear resistance in our previous study [8]. Therefore, Al_1.8_CrCuFeNi_2_ HEA was selected as the main coating material. The 200 mesh Al_1.8_CrCuFeNi_2_ HEA powders prefabricated using an aerosol method (Linyi Research and Innovation Material Technology Co., Ltd., Linyi, China.) were used.

TiC was selected as the strengthening phase due to its extremely high hardness (above 3000 HV) and low density (4.93 g/cm^3^). Its excellent microstructure makes it a high-quality nucleation core in the alloy system to promote grain refinement. Moreover, the high hardness and high melting point of TiC particles enable them to be pinned between grains, which plays the role of second-phase strengthening and greatly improves the hardness and wear resistance of the material [20].

The laser cladding experiment was carried out using the 3D rapid prototyping scanning remanufacturing repair system. The machine parameters are shown in Table 1. Firstly, the powder was dried at 100 °C for 30 min to prevent water in the powder from affecting the experimental results and increase the fluidity of the powder. Then, the powder was put into the powder feeder for standby. The powder feeder adopted roller-type powder feeding. The powder feeding speed was controlled by adjusting the rotating speed of the powder feeder. The rotating speed was 1.3 r/min, and the corresponding powder feeding speed was about 8 g/min. Argon was used as a protective gas in the process of powder feeding, and the powder was transported through the flow of argon.

### 2.2. Structure Analysis

The phase structure was analyzed using an X-ray diffractometer (XRD, empyrean) from the Netherlands. The working voltage was 35 kV, the working current was 50 mA, the scanning speed was 4°/min and the scanning angle was 20–100°.

The microstructure was analyzed using an energy dispersive spectrometer (X-MAX 50) equipped with an electron microscope.

### 2.3. Analysis of Hardness and Wear Resistance

The microhardness was measured using a tmvp-1 hardness tester. Five areas on the sample surface and four hardness values in each region were selected to measure the hardness. The trim mean was the final hardness value.

The wear resistance of the samples was measured using a mms-2a friction and wear testing machine under the conditions of dry friction and room temperature. The experimental load was 100 N, and the test time was 0.5 h and 2 h, respectively.

## 3. Results and Discussions

**Figure 1:**
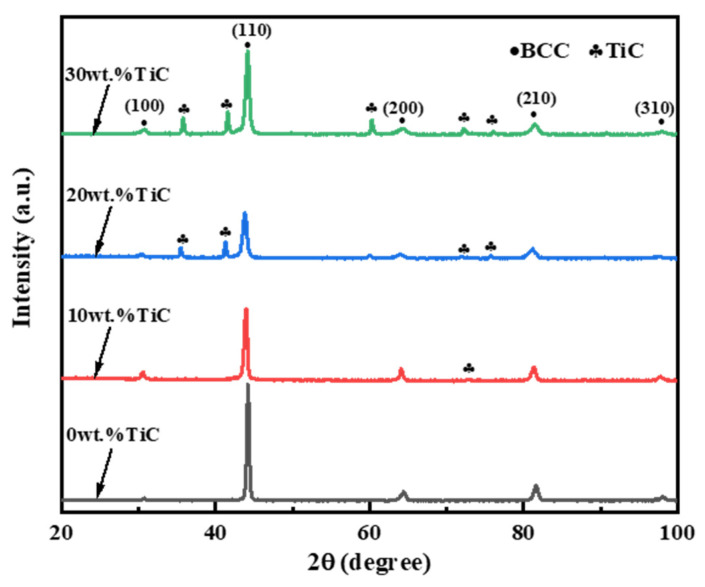
XRD patterns of the coatings with different TiC contents.

Figure 1 shows the XRD diffraction patterns in the Al_1.8_CrCuFeNi_2_ HEA-based coatings with different TiC contents. The results showed that a single solid solution of BCC was formed in the Al_1.8_CrCuFeNi_2_ HEA, which was consistent with previous research [15]. After adding 10 wt.% TiC, the phase structure of the coating was still the single BCC phase. No obvious peaks in the TiC phase diffraction were found, which was similar to the report in [19]. In addition, the diffraction peak was slender, indicating that the crystal crystallinity was high and the grain size was large [21]. When 20 wt.% TiC was added, the half-height width of the BCC phase diffraction peak increased significantly. The results indicated that the crystallinity and grain size of the coating decreased. The main reason was that after too much TiC was added to the grains in the crystal, the TiC was dispersed in the coating, which hindered the movement of the grain boundaries and inhibited the growth of the grains. In addition, when the content of TiC was increased to 20 wt.%, some new diffraction peaks corresponding to the TiC compound appeared in the XRD diffraction pattern. The reason was that with the increase in the TiC content, the TiC in the grain was gradually saturated, and a large number of TiC-agglomerated particles were formed. When the TiC content increased to 30 wt.%, the height of the diffraction peak in the BCC structure increased, but the half-height width did not obviously narrow. Therefore, according to the Scherrer formula, the grain size in the BCC phase did not change significantly, but the content in the BCC phase increased [22].

**Figure 2:**
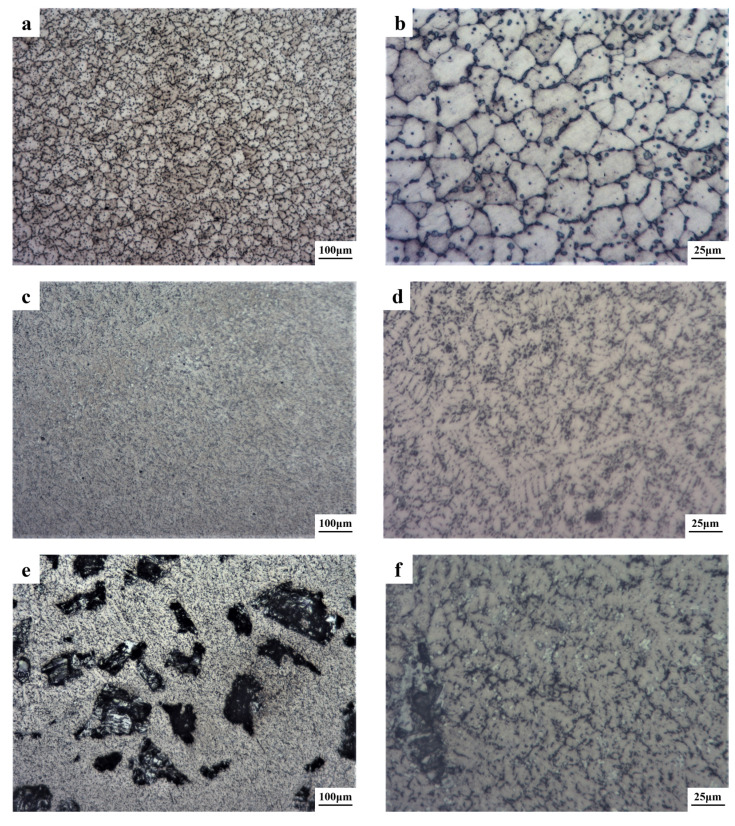

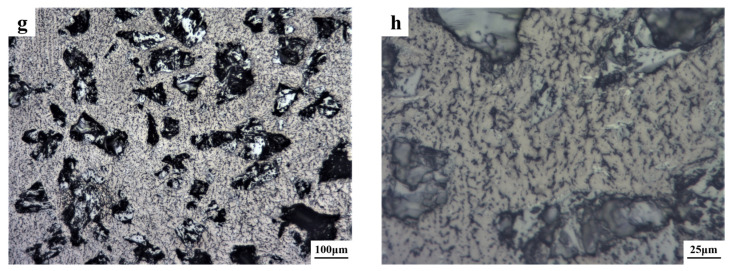
Metallographic structure of high-entropy alloys with different TiC contents: (**a**,**b**) 0 wt.%; (**c**,**d**) 10 wt.% TiC; (**e**,**f**) 20 wt.% TiC; (**g**,**h**) 30 wt.% TiC.

Figure 2 shows the metallographic structure of the coatings. From the metallographic diagram, the crystalline grain of the coating was obviously refined after the TiC was added. When the content of the TiC was 10 wt.%, the grain was evenly distributed without other precipitates. However, when the content of the TiC increased to 20 wt.%, the large agglomerated particles appeared, the shape of the precipitates was dendritic-like and the distribution was relatively concentrated. When the TiC content increased to 30 wt.%, the size of the agglomerated plaque in the particles decreased, but its quantity increased significantly.

**Figure 3:**
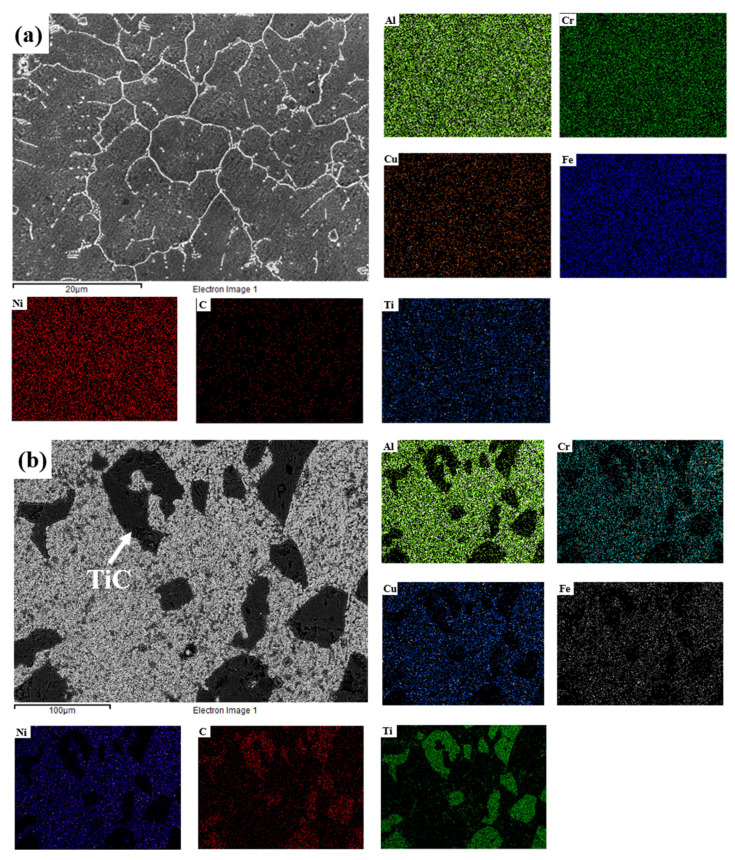

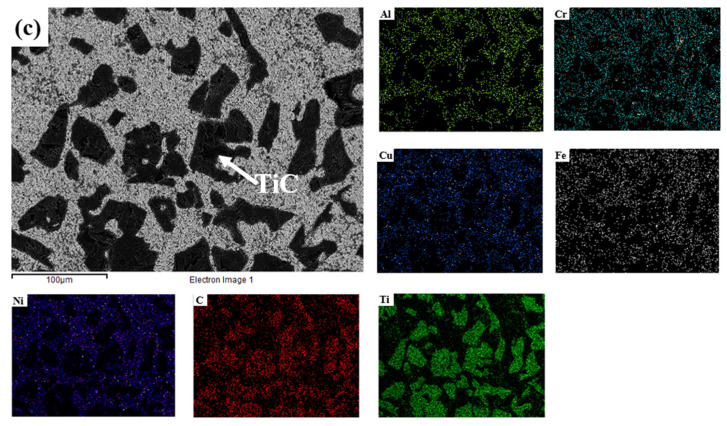
EDS mapping results of the coatings with different contents of TiC: (**a**) 10 wt.% TiC; (**b**) 20 wt.% TiC; (**c**) 30 wt.% TiC.

The results of the EDS mapping of the coatings are shown in Figure 3. In the 10 wt.% TiC coating, the elements were uniformly distributed. In the black agglomerated particle area in the 20 wt.% TiC and 30 wt.% TiC coatings, the content of Ti and C was aggregated, and other elements were less aggregated. This showed that the large agglomerated particles were TiC. In addition, the other elements were uniformly distributed in the area outside of the TiC-agglomerated particles.

It could be seen from the high-magnification metallographic diagram that when the TiC content increased from 10 wt.% to 20 wt.%, the amount of intergranular structure in the coatings increased, the color became darker and the shape of the grain changed from columnar dendrite to small dendrite. When the TiC content increased to 30 wt.%, the distribution of the grain was more regular than that of the 20 wt.%, and the grains gradually became columnar dendrite, which was mainly related to the increase in the TiC content. When the TiC content was 10 wt.%, the TiC dissolved in the high-entropy alloy to form the BCC phase. However, when the content increased to 20 wt.%, the TiC content dissolving in the high-entropy alloy was too high, resulting in the formation of the agglomerated particles, which was TiC. Moreover, with the increase in the TiC content to 30 wt.%, more TiC-agglomerated particles were formed.

**Figure 4:**
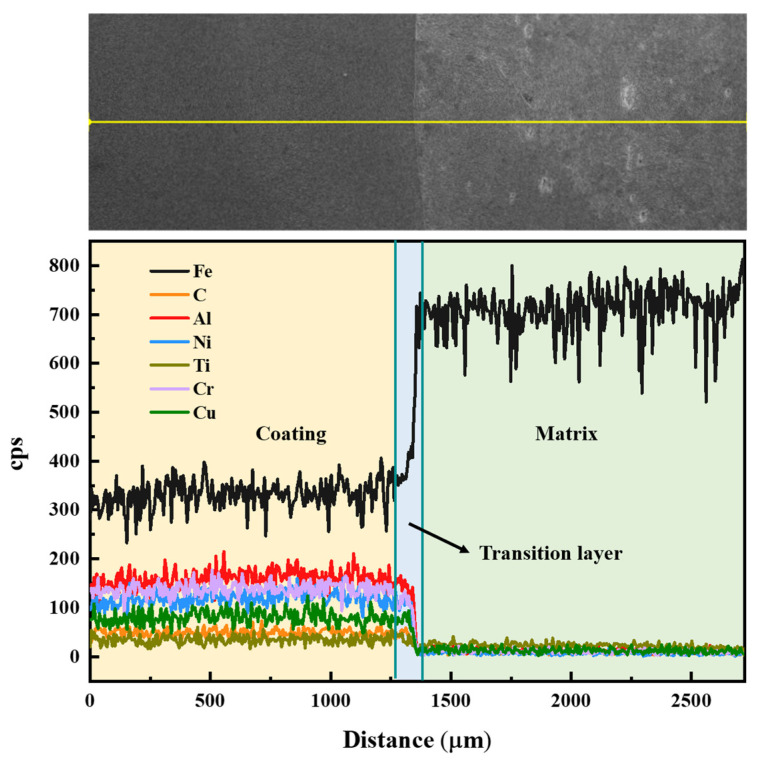
Element distribution between coating and substrate.

Figure 4 shows the longitudinal section morphology and element distribution of the matrix, transition layer and coating with 10 wt.% TiC. The three regions are clearly defined in the figure. The element distribution was analyzed from the substrate to the coating. From the matrix to the transition layer, the elements showed an obvious diffusion trend, which indicated that the formation of the molten pool enabled the movement and diffusion of each element atom during the cladding process, and the heat provided the kinetic energy for the atom movement. Therefore, the element bonding state was excellent, and could maintain perfect metallurgical bonding. In addition, Ti and C were uniformly dispersed in the coating, which increased the overall hardness of the coating.

**Figure 5:**
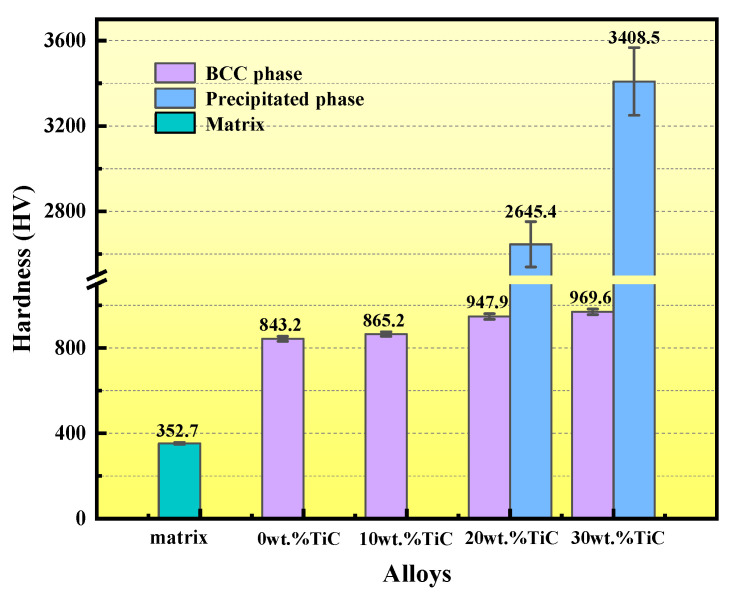
The hardness of different alloys and the relative lifting range of matrix.

Figure 5 shows the hardness of the matrix and composite coatings with different TiC contents. The results showed that the hardness of the high-entropy alloy coating was greatly improved compared with the matrix. Moreover, with the increase in the TiC content, the hardness of the BCC phase and the TiC-agglomerated particles in the composite coating increased continuously.

In order to further investigate the hardness of the coatings, the TiC-agglomerated particles were also studied. Figure 4 shows the microhardness values of the TiC-agglomerated particles in the coatings. Only when the content of TiC was more than 10 wt.%, would the new phase precipitate. The hardness value of the TiC-agglomerated particles was very high. According to Figure 2, with the increase in the TiC, the TiC-agglomerated particles increased and became evenly distributed, which could greatly increase the properties of the coating.

The increase in the hardness of the coating was related to two strengthening mechanisms, which were dispersion strengthening and grain refinement [16,17,23]. Dispersion strengthening is a strengthening mechanism by which a large number of other external phases are added to the high-entropy alloy. As a result, the content of the phase in the high-entropy alloy far exceeds its solubility, thus dispersing the structure of other phases. TiC existed in the form of large-sized agglomerated particles in the coating, which bore the main load. This method belonged to load strengthening. Because the coefficient of thermal expansion of the TiC and HEA did not match, the dislocation density of the coating would increase. This phenomenon improved the overall performance of the coating [16,18,24]. Grain refinement is a common strengthening mechanism. It mainly affects the grain size of the original composition by adding particles. The TiC particles would preferentially form cores in the high-entropy alloy systems, consuming a large amount of grain boundary energy to hinder the grain growth [17]. The increase in the grain boundary density inevitably led to the accumulation of dislocations, thereby improving the overall hardness of the coating.

Table 2 shows the wear loss of the matrix and coatings after the friction and wear tests for 30 min and 120 min. The results showed that the wear loss of the coating was obviously lower than that of the substrate. Furthermore, with the increase in TiC content, the wear loss of the coating decreased. In particular, when the TiC content exceeded 20 wt.%, the wear loss of the coating obviously decreased and the wear resistance was greatly improved. The above results were consistent with Archard’s law: the wear resistance of the material is positively correlated with surface hardness, and the wear loss is negatively correlated with surface hardness [25].

In this study, with the increase in TiC, the hardness increased and the wear loss decreased. Therefore, the coating with 30 wt.% TiC not only exhibited a high hardness, but also presented a low wear loss, which indicated that the coating with 30 wt.% TiC exhibited the best comprehensive performance among the coatings studied.

**Figure 6:**
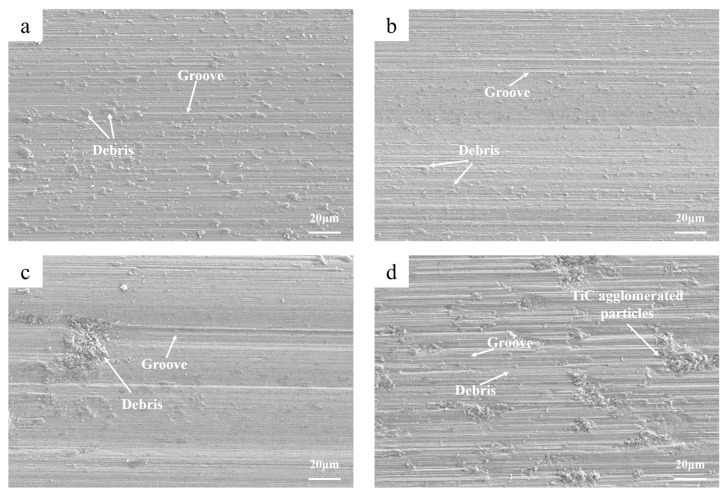
**Figure 6.** Wear morphology of reinforced coatings with different contents of TiC: (**a**) 0 wt.% TiC; (**b**) 10 wt.% TiC; (**c**) 20 wt.% TiC; (**d**) 30 wt.% TiC.

Figure 6 shows the wear morphology of the coatings with different contents of TiC. The wear forms of the coatings with the different TiC contents were obvious abrasive wear. The coating was a brittle material, which was mainly damaged by the normal force from the friction test ring during the friction process. Combined with the metallographic diagram (Figure 1) and the XRD diffraction pattern (Figure 2) analysis, it was found that there were obvious agglomerated particles in the coating with a more than 20 wt.% TiC content. This was also the main reason why the wear loss in the coating with 20 wt.% and 30 wt.% TiC decreased greatly. On the one hand, with the increase in TiC content, the number of TiC-agglomerated particles increased. Due to the ultra-high hardness of TiC, it played a load-bearing role in the friction and wear process and was not easy to be destroyed by normal. On the other hand, with the increase in TiC content, the overall hardness of the coating increased, and the ability of the whole surface to resist normal damage increased. There was more wear debris and deeper grooves on the surface of the 0 wt.% TiC coating. The wear debris on the surface of the coating with 10 wt.% TiC was reduced and the groove was also shallow. With the further increase in TiC content, the groove became deeper. The reason is that compared with the 0 wt.% TiC coating, the hardness of the coating after adding 10 wt.% TiC was improved, and no TiC-agglomerated particles appeared, resulting in less wear debris, and the grooves drawn by the wear debris on the surface were also shallow. A large number of TiC-agglomerated particles appeared in the 20 wt.% TiC and 30 wt.% TiC coatings. During the friction and wear process, some hard TiC was destroyed and fell from the coating to form debris. The TiC debris was deeper than the grooves scratched by the high-entropy alloy debris on the coating.

## 4. Conclusions

In this study, the TiC-reinforced Al_1.8_CrCuFeNi_2_ HEA coatings were fabricated onto the 65 Mn surface using laser cladding. The influence of TiC on the structure and wear resistance of the coatings was studied. The main conclusions could be summarized as follows:

(1) A single solid solution of BCC was formed in the Al_1.8_CrCuFeNi_2_ HEA. The 10 wt.% TiC coating was still a single solid solution of BCC without obvious TiC agglomeration particles. The TiC-agglomerated particles appeared in both the 20 wt.% TiC and the 30 wt.% TiC coating. After adding TiC, the grain size clearly decreased.
(2) Due to the fine-grain strengthening and dispersion strengthening of TiC, with the increase in TiC content, the hardness of the high-entropy alloy coatings clearly increased. The hardness of the 30 wt.% TiC coating was the highest.
(3) The wear resistance of the Al_1.8_CrCuFeNi_2_ HEA coatings could be significantly enhanced with the addition of TiC. Due to the increase in hardness, the 30 wt.% TiC/Al_1.8_CrCuFeNi_2_ composite coating had the best wear resistance.

## Figures and Tables

**Table 1:** Parameters of laser cladding equipment.

Content	Model/Parameter
Laser	YLS-4000
Laser cladding head	YC52
Powder feeder	AFS-PF-D
Six-axis industrial robot	KR30HA
Double-axis turntable	KUKA DKP400
Vertical and horizontal single-axis turntable	HR-630R
Mobile workbench	1500\times1000
Inert gas purification system	GP400

**Table 2:** Wear loss of 65 Mn and TiC/high-entropy alloy coatings (mg).

Time	65 Mn	0 wt.% TiC	10 wt.% TiC	20 wt.% TiC	30 wt.% TiC
30 min	21.6 ± 1.3	11.7 ± 0.5	11.3 ± 0.5	2.1 ± 0.1	2.2 ± 0.1
120 min	38.1 ± 2.1	37.1 ± 1.5	36.5 ± 0.7	9.1 ± 0.2	8.1 ± 0.4

## Data Availability

All data that were generated or analyzed during this study are included in the article.
